# Reductions in US life expectancy from COVID-19 by Race and Ethnicity: Is 2021 a repetition of 2020?

**DOI:** 10.1101/2021.10.17.21265117

**Published:** 2021-12-17

**Authors:** Theresa Andrasfay, Noreen Goldman

**Affiliations:** a Leonard Davis School of Gerontology, University of Southern California; b Office of Population Research, School of Public and International Affairs, Princeton University

## Abstract

COVID-19 had a huge mortality impact in the US in 2020 and accounted for the majority of the 1.5-year reduction in 2020 life expectancy at birth. There were also extensive racial/ethnic disparities in the mortality impact of COVID-19 in 2020, with the Black and Latino populations experiencing reductions in life expectancy at birth over twice the reduction experienced by the White population. Despite continued vulnerability of the Black and Latino populations, the hope was that widespread distribution of effective vaccines would mitigate the overall impact and reduce racial/ethnic disparities in 2021. In this study, we use cause-deleted life table methods to estimate the impact of COVID-19 mortality on 2021 US period life expectancy. Our partial-year estimates, based on provisional COVID-19 deaths for January-early December 2021 suggest that racial/ethnic disparities have persisted and that life expectancy at birth in 2021 has already declined by 1.5 years from pre-pandemic levels. Our projected full-year estimates, based on projections of COVID-19 deaths through the end of 2021 from the Institute for Health Metrics and Evaluation, suggest a 1.9-year reduction in US life expectancy at birth from pre-pandemic levels, a steeper decline than the estimates produced for 2020. The reductions in life expectancy at birth estimated for the Black and Latino populations are 1.4–2.1 times the impact for the White population.

## Introduction

**Figure 1:**
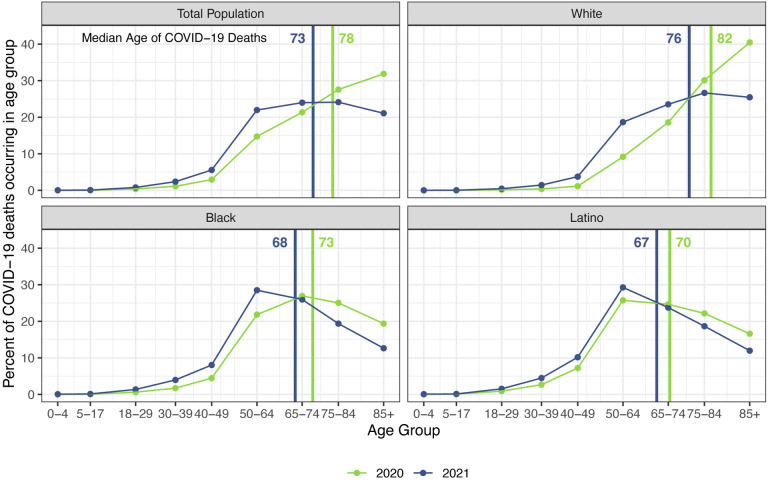
Percentage of COVID-19 deaths in each age group: 2020 vs. 2021. Data are from provisional COVID-19 deaths through December 4, 2021, provided by the National Center for Health Statistics (December 8, 2021 update).

The staggering death toll in the US from COVID-19 has been well-documented: deaths attributed to COVID-19 in 2020 account for almost three-quarters of the 1.5-year reduction in period life expectancy at birth, reversing over 16 years of progress in mortality improvement (1). In a previous paper, we predicted that widespread availability of an effective vaccine would lessen the impact of COVID-19 on 2021 life expectancy compared with 2020, although life expectancy was unlikely to return to pre-pandemic levels (2). Several highly effective vaccines have indeed been developed in record time, but relatively low vaccine coverage in the US, combined with the highly transmissible Delta variant of SARS-CoV-2, have led to a fourth mortality surge, and by late November, the total number of COVID-19 deaths in 2021 had already exceeded the 2020 total (3, 4). These sobering numbers combined with the younger age distribution of deaths in 2021 (see Figure 1) – resulting partly from higher vaccination rates among older individuals – indicate that the impact of COVID-19 on life expectancy in 2021 will be larger than that in the preceding year.

The disproportionate impact of COVID-19 on the survival of vulnerable populations has also received extensive attention: the Latino and Black populations experienced declines in life expectancy over twice as large as that for Whites (1). Risk factors for COVID-19 infection and mortality, such as crowded living conditions, frontline jobs with high exposure to infection and low pay, dependence on public transport, low access to quality healthcare, and high rates of select chronic conditions, still characterize these groups, suggesting continued racial/ethnic disparities in COVID-19 mortality (5–9). A strategically-targeted vaccine distribution had the potential to reduce racial/ethnic disparities in COVID-19 mortality in 2021 (10), but many individuals faced barriers to vaccination in the early months, including difficulty scheduling vaccine appointments online, lack of transportation to vaccination sites, and lack of time off work to get vaccinated and recover from side effects (11, 12). The resulting inequitable vaccine distribution and uptake may have further exacerbated racial/ethnic disparities in COVID-19 mortality. However, as vaccines became more widely available in the later months of 2021, racial/ethnic differentials in vaccination rates decreased (13, 14). By August 2021, differences in vaccination rates by political affiliation, religion, and rural/urban status exceeded racial/ethnic differences (13), suggesting a potential reduction of racial/ethnic disparities in COVID-19 mortality in 2021 relative to those in 2020.

In the current study, we extend our previous work by estimating the impact of COVID-19 on period life expectancy at birth and at age 65 in 2021 for the total population and for the White, Latino, and Black populations in the US. We present estimates for the partial year (through December 4, 2021) as well as for all of 2021 using projected numbers of COVID-19 deaths from the Institute for Health Metrics and Evaluation for the remainder of the year.

## Methods

To estimate the impact of COVID-19 on 2021 period life expectancy (*e*_*x*_) with partial-year data on all-cause mortality, we employ life table techniques developed to estimate the impact on life expectancy of eliminating one or more causes of death (i.e., cause-deleted life tables; Chiang, 1968). In the present study, we assume that mortality conditions in 2021 would be equivalent to those observed pre-pandemic (i.e., had COVID-19 not occurred), and then estimate how the inclusion of COVID-19 deaths alters these mortality conditions. Specifically, we take the pre-pandemic life tables to be cause-deleted life tables in which COVID-19 has been eliminated and recover all-cause life tables for 2021 that incorporate COVID-19 mortality. This strategy has been used in previous studies to estimate the impact of COVID-19 on 2020 life expectancy (2, 15–17). Although this procedure does not incorporate excess mortality from causes other than COVID-19, our life expectancy at birth estimate of 1.3 years for the total US population based on this method (15) was within 0.2-years of the official 1.5-year National Vital Statistics System (NVSS) estimate (1).

Provisional COVID-19 deaths by age, race, and ethnicity are provided by the National Center for Health Statistics (18). These data include all deaths for which COVID-19 is listed as an underlying, probable, or presumed cause of death, and include deaths through December 4, 2021. The projected total number of COVID-19 deaths in the US by December 31, 2021, are taken from the November 17, 2021 projections issued by the Institute for Health Metrics and Evaluation (IHME) (19). At the time of writing, IHME published three scenarios for total reported COVID-19 deaths; we take the medium “current projection scenario” for our analyses. Because IHME projects cumulative COVID-19 deaths since the beginning of the pandemic, we subtract the total number of COVID-19 deaths reported by December 31, 2020 to obtain a total projected death toll for 2021.^1^ Mid-year 2021 population estimates by age, race, and ethnicity are obtained from the US Census Bureau (20). Life tables for 2018 for the total US population and for the non-Latino White, non-Latino Black, and Latino populations are obtained from the NVSS (21).^2^

We first estimate the expected number of deaths in 2021 in the absence of COVID-19 (_n_D_x,2021_*) by multiplying the 2018 age-specific mortality rates (_n_Mx,2018) by the 2021 mid-year population for the same age range (_n_K_x,2021_). We then estimate the expected number of deaths in 2021 in the presence of COVID-19 (_n_D_x,2021_) by adding the number of COVID-19 deaths in each age group (_n_COV_x,2021_) to the expected number of deaths in each age group from other causes. We assume that individuals who do not die of COVID-19 in 2021 are exposed to the 2018 mortality risks:

nDx,2021*=Mnx,2018*Knx,2021nDx,2021=CnOVx,2021+Mnx,2018*(nKx,2021−CnOVx,2021)

We then calculate the age-specific ratio of expected number of deaths in the absence of COVID-19 to expected number of deaths in the presence of COVID-19 (_n_R_x,2021_). Using this ratio and Chiang’s method (22), we adjust the 2018 life table values to reflect the presence of COVID-19 mortality and obtain our estimates of all-cause life tables for 2021. We repeat these calculations for each of the racial/ethnic groups in our study.

Since total COVID-19 deaths in 2021 are still unknown at the time of writing (December 2021), we make two sets of calculations for 2021. The first set provides *partial-year* estimates that are based only on COVID-19 deaths reported to the NCHS through December 4, 2021. These calculations implicitly assume that there will be no additional COVID-19 deaths in 2021; they should be interpreted as an estimate of how COVID-19 deaths in 2021 *thus far* have impacted life expectancy and are a lower bound on the final impact in 2021. The second set provides estimates based on the total number of COVID-19 deaths projected by IHME under their “current projection scenario”, which is their medium projection. Given that IHME does not project deaths separately by age and race/ethnicity, we assume that future COVID-19 deaths in 2021 will have the same age and racial/ethnic distribution as deaths that have already occurred in 2021.

Because our cause-deleted life table methodology differs from that used by NVSS to estimate 2020 life expectancy, the magnitude of our 2021 estimates will not be directly comparable to the published 2020 life expectancy estimates from NVSS. To facilitate comparisons between 2020 and 2021, we estimate 2020 life expectancy with the same methods as our 2021 calculations, using the provisional counts of COVID-19 deaths (rather than deaths from all causes) provided by NCHS for all of 2020, and mid-year 2020 population estimates provided by the US Census Bureau.^3^

## Results

**Figure 2:**
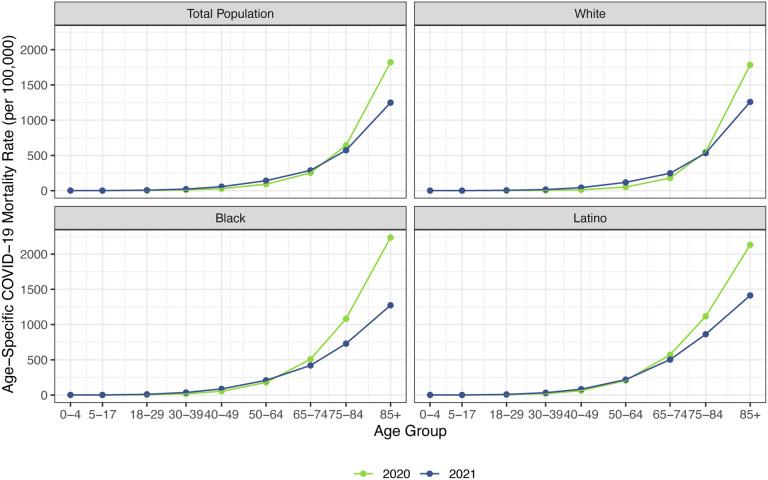
Age-specific COVID-19 mortality rates in 2020 and 2021 by race/ethnicity. 2020 estimates are based on NCHS provisional counts of COVID-19 deaths for all of 2020. 2021 estimates are based on NCHS provisional counts of COVID-19 deaths through December 4, 2021.

**Figure 3:**
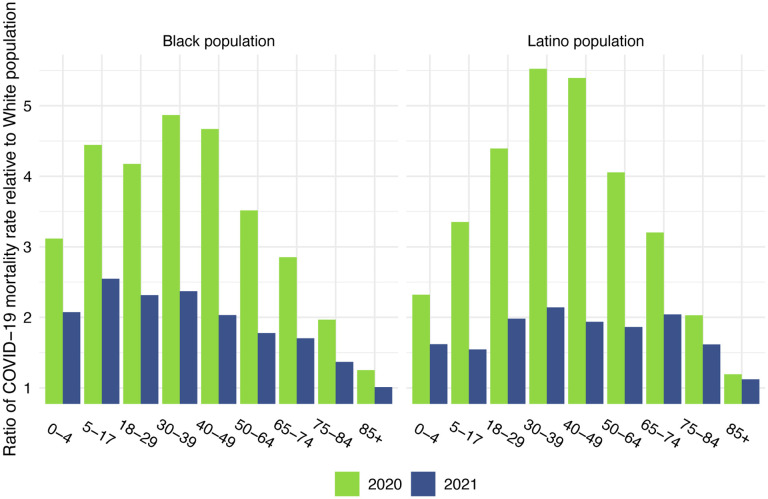
Ratio of age-specific COVID-19 mortality rate relative to the White population. 2020 estimates are based on NCHS provisional counts of COVID-19 deaths for all of 2020. 2021 estimates are based on NCHS provisional counts of COVID-19 deaths through December 4, 2021.

Before presenting the estimates of life expectancy during the pandemic, we display descriptive statistics of the age-specific COVID-19 mortality rates, which are defined as the number of COVID-19 deaths in an age group in a given year divided by the total population in that age group at the mid-point of the year. Figure 2, which presents age-specific COVID-19 death rates for the past two years, portrays a similar pattern across racial/ethnic groups: a large decline in rates at the oldest ages between 2020 and 2021, with modest changes – often small increases – below age 65. Figure 3, which shows these rates for the Black and Latino populations divided by the corresponding rates for the White population, highlights the huge decline in relative death rates for the working age population (18–65): COVID-19 death rates at these ages were often three to five times as high in the Black and Latino populations as among Whites in 2020 but roughly twice as high in 2021. These numbers suggest a substantial decline in racial/ethnic disparities in COVID-19 mortality from 2020 to 2021, but the estimates of life expectancy provided below tell a more nuanced and sobering story.

**Figure 4:**
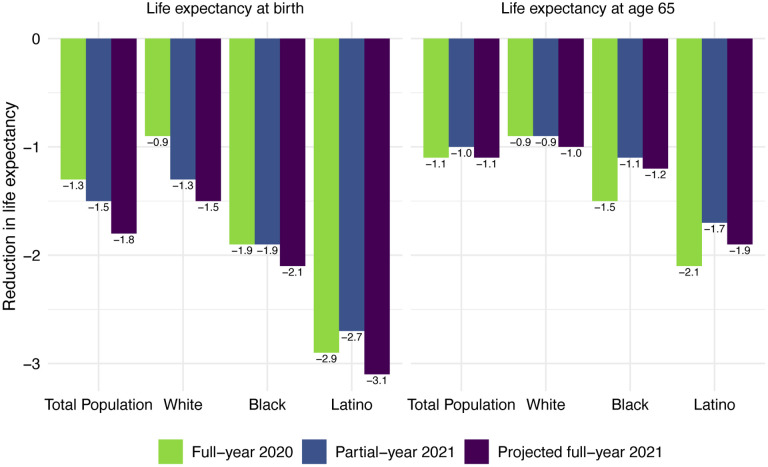
Reduction in life expectancy at birth due to COVID-19 mortality by race/ethnicity and by year. Changes are all relative to 2018 life expectancy. 2020 estimates are based on NCHS provisional counts of COVID-19 deaths for all of 2020. 2021 partial-year estimates are based on NCHS provisional counts of COVID-19 deaths through December 4, 2021. 2021 full-year projections are based on the projected number of total reported COVID-19 deaths through December 31, 2021 (November 17, 2021 update).

Life expectancy estimates for the total US population and by race/ethnicity are displayed in Table 1. All reductions are relative to 2018 life expectancy values, which are displayed in panel A at the top of this table. Our estimates of 2020 life expectancy reductions due to COVID-19 are displayed in panel B; these are estimated using cause-deleted methods, the same procedure used for the 2021 estimates. To highlight the comparisons between 2020 and 2021 under each of these scenarios, Figure 4 displays the magnitudes of the reductions in life expectancy at birth and at age 65 for the full-year 2020 estimates, partial-year 2021 estimates, and projected full-year 2021 estimates.

Panel C of Table 1 displays the partial-year 2021 estimates, based on the number of COVID-19 deaths through December 4, 2021. These estimates indicate that COVID-19 deaths through the first eleven months of 2021 already imply a 1.5-year reduction in life expectancy at birth and a 1.0-year reduction in life expectancy at age 65 for the total US population. The reductions in life expectancy at birth are largest for the Latino population (2.7 years), followed by the Black population (1.9 years), and smallest for the White population (1.3 years). The reductions in life expectancy at birth for the total and White populations already exceed the reductions estimated for the full year in 2020. The 2021 partial-year reduction in life expectancy at birth for the Black population is equivalent to the 2020 reduction, while for the Latino population the 2021 partial-year reduction is 0.2 years lower than that of 2020.

Panel D of Table 1 presents the 2021 estimates for a full-year based on the total number of reported COVID-19 deaths projected by IHME on November 17, 2021. Under this projection of 456,957 total reported COVID-19 deaths in 2021, there would be a 1.9-year reduction in life expectancy at birth for the total US population, which substantially exceeds the 1.3-year estimated reduction for 2020 due to COVID-19. The 1.1-year reduction in life expectancy at age 65 for the total US population is equal to that estimated for 2020. These estimates for the reduction in life expectancy at birth in 2021 *exceed those for 2020 for all three racial/ethnic groups*, with the difference between 2021 and 2020 declines largest for the White population (an additional 0.6-year decline). The estimated reductions in life expectancy at birth for the Latino population (3.1 years) and the Black population (2.1 years) are 2.1 and 1.4 times, respectively, the 1.5-year projected reduction for the White population. Although these estimates of loss in life expectancy relative to Whites are below those in 2020, the disparities have narrowed far less than suggested by the large decreases in relative age-specific death rates between 2020 and 2021 shown in Figure 3. This seeming inconsistency between the magnitude of relative death rates and the magnitude of the corresponding life table comparisons appears in various demographic contexts (23). Regardless of the metric, these disparities reveal another year of egregious racial/ethnic inequities underlying a large overall impact of COVID-19 on life expectancy.

## Discussion

Our preliminary estimates of period life expectancy suggest a devasting impact of COVID-19 in 2021, one that will be larger than that in 2020. As with our 2020 estimates (15), we project that the Latino population will experience the largest reduction in life expectancy at birth in 2021 due to COVID-19, perhaps as much as one year higher than the Black population. The overall impacts on life expectancy in 2021 will likely be even greater than those shown here because our estimates incorporate deaths from only COVID-19. The effect of omitting net increases in numbers of deaths from other causes in 2020 is apparent from the NVSS estimates for life expectancy, which slightly exceed our estimates for 2020 based only on COVID-19 deaths (1, 15).

These discrepancies with the NVSS estimates for 2020 arise primarily from the unrealistic assumption of independence underlying the cause-deleted procedure: i.e., that the introduction of COVID-19 did not alter the risks of dying from other conditions. A comparison of cause-specific death rates between 2019 and 2020 indicates a net rise in mortality from non-COVID-19 causes in 2020, often referred to as “excess” deaths: increases in several causes (e.g., drug overdoses and other unintentional injuries, homicides, diabetes) had a larger overall impact on life expectancy than decreases in other causes (e.g., cancer, Alzheimer’s disease, influenza, chronic lower respiratory diseases) (1, 24). Because the risk of COVID-19 fatality is increased in the presence of numerous co-morbidities (e.g., cancer, Alzheimer’s disease), mortality rates from some of these chronic diseases likely decreased as severely ill patients, particularly those with compromised immune systems, succumbed to COVID-19 rather than their underlying condition. Increased mortality rates from other non-COVID-19 causes may have resulted from delays in primary and preventive care or reduced disease management, as well as from inadequate healthcare due to shortages of equipment, staff, and space (25–28). Increased risks of dying from a broad range of conditions may also have been triggered by detrimental changes in health-related behaviors induced by the many social and economic stressors during the pandemic; these behaviors include higher rates of smoking, drinking and drug use; worse nutrition; and reduced exercise (29–31).^4^ It is possible that the impact of the pandemic on non-COVID-19 mortality will be lessened in 2021 as many healthcare facilities have resumed close to pre-pandemic levels of operation and some shortages of personnel and supplies have been resolved, but this could be counteracted by elevated mortality among those who have recovered from COVID-19 (34). Finally, misidentification or miscoding of cause of death could have contributed to either increases or decreases in non-COVID-19 causes of death. These errors should have been reduced in 2021 because of increased availability of COVID-19 diagnostic tests.

Our estimates in Table 1 reveal little difference between projected declines in life expectancy at age 65 for 2021 and those estimated for 2020. This comparison contrasts with the larger declines in life expectancy *at birth* projected for 2021 compared with those estimated for 2020 for all groups. These patterns reflect the shifting distribution of ages at death toward younger ages in 2021 (Figure 1). This is likely due in large part to the steady increase in the prevalence of vaccination by age with high levels achieved for the elderly (over 85% of the 65 and over population fully vaccinated by early December 2021), in contrast to lower coverage among younger adults (approximately 62% of adults aged 25–39 fully vaccinated by early December 2021) (3). High vaccination rates among nursing home residents, who are particularly vulnerable to adverse COVID-19 outcomes, paired with stricter infection protocols, also helped reduce the mortality impact of COVID-19 on older adults in 2021 (35, 36). The net result, as shown in Figure 2, is that mortality from COVID-19 for each racial/ethnic group has declined substantially at the oldest ages, particularly for the Black and Latino populations, but has changed relatively little at young and middle ages. Although differences between 2020 and 2021 in age-specific death rates at young and middle ages appear small in Figure 2, the modest increases throughout much of this age range, most notable for Whites, contribute to the greater reductions in life expectancy at birth in 2021.

Our estimates underscore the continued large racial/ethnic disparities in the impact of COVID-19 on life expectancy. The disproportionately high losses of life in the Black and Latino populations reflect the social and economic inequities that have been repeatedly acknowledged throughout the pandemic, most notably high rates of poverty and crowded housing, low income jobs that cannot be performed remotely, a high prevalence of chronic health conditions, and inadequate access to quality healthcare (5, 6, 8). Latinos, who once again appear to have suffered the greatest loss of life from COVID-19, have particularly low levels of health insurance coverage, are more likely to live in multigenerational households than most other groups, and often face language barriers to obtaining comprehensible information on viral transmission and mitigation strategies (6, 7, 9, 37). In addition, Latino workers suffered disproportionate job and income losses during the pandemic because of their overrepresentation in the gig economy and in industries greatly impacted during this period (e.g., construction and leisure and hospitality) and because many Latinos were ineligible for government benefits (38). Although data on race and ethnicity of vaccine recipients are incomplete, existing information suggests that the persistent racial/ethnic disparities are likely partially the result of differences in vaccine uptake early in 2021. For example, after taking differences in age structure into account, Reitsma and colleagues estimate that vaccine uptake rates (for at least one dose) were about 30% higher in Whites than in the Black and Latino populations through the end of March, 2021, with huge variability across states (39).

Although the estimates of life expectancy decline in Table 1 indicate some narrowing of the differentials from the previous year, this is entirely due to larger life expectancy reductions in the White population rather than to smaller decreases in either the Black or Latino populations. The recent worsening of COVID-19 mortality among Whites could reflect lower adherence to social distancing guidelines relative to other races/ethnicities (40, 41).

This analysis is subject to several limitations. As previously mentioned, the cause-deleted life table methodology does not account for excess mortality from causes other than COVID-19. Our partial-year estimates for 2021 life expectancy rely on NCHS provisional COVID-19 deaths, which are subject to reporting and processing delays, and so they are likely to be an underestimate of the impact of COVID-19 on life expectancy thus far in 2021. Our estimates for the full 2021 year rely on projected deaths for the remainder of 2021, which will depend on further vaccine uptake, waning vaccine efficacy, the severity and spread of the Omicron variant, and the magnitude of the current winter surge in cases, among other factors. We will update these estimates when the 2021 data on COVID-19 deaths become complete.

As period measures, life expectancy estimates for 2021 summarize the mortality conditions of 2021 and do not represent expectations of remaining life for any living cohort, which will depend on future mortality conditions. It is uncertain whether mortality conditions, and thus life expectancy, in 2022 will return to pre-pandemic levels. There are several reasons for optimism related to COVID-19 mortality in 2022. The recent expansion of vaccine eligibility to children ages 5 and up should help slow transmission of the SARS-CoV-2 virus in the population, while the administration of booster doses should help protect those most at risk of dying from complications of COVID-19 (42, 43). Other efforts that should reduce the mortality impact of COVID-19 are the ongoing development of different types of vaccines, including those targeting new variants, and of effective oral antiviral treatments, which will be easy to administer and reduce the risk that a COVID-19 infection develops into severe disease (44).

However, there are also several reasons why 2022 may see continued elevated mortality levels and persistent inequities. There is still substantial vaccine refusal in the US, with approximately 20% of adults not having received any dose of a COVID-19 vaccine by early December 2021 (3). As with the recent appearance of the easily transmissible Omicron variant, there is a constant threat of the emergence of new variants of SARS-CoV-2 that are at least partly resistant to existing vaccines. There is also evidence that survivors of COVID-19 have increased mortality risks for at least six months following initial recovery (45), and the mortality impact of long COVID is not yet known. And, there likely will be other long-term impacts of the pandemic on mortality resulting from the many social, economic, and healthcare disruptions during the past two years that will continue to disproportionately affect vulnerable populations.

## Figures and Tables

**Table 1:** Life expectancy estimates and reductions from 2018 for the total US population and by race/ethnicity

	Total Population		non-Latino White		non-Latino Black		Latino	
	Birth	Age 65	Birth	Age 65	Birth	Age 65	Birth	Age 65
**A) Pre-pandemic**								
2018 e_x_	78.7	19.5	78.6	19.4	74.7	18.0	81.8	21.4

**B) Full-year 2020 estimates**								
Number of COVID-19 deaths	385,348		232,770		61,481		69,468	
Estimated 2020 e_x_	77.4	18.4	77.7	18.5	72.8	16.5	78.9	19.3
Reduction from 2018 e_x_ due to COVID-19	−1.3	−1.1	−0.9	−0.9	−1.9	−1.5	−2.9	−2.1

**C) Partial-year 2021 estimates**								
Number of COVID-19 deaths through December 4, 2021	402,919		260,862		55,031		66,666	
Estimated 2021 e_x_	77.2	18.5	77.3	18.5	72.8	16.9	79.1	19.7
Reduction from 2018 e_x_ due to COVID-19	−1.5	−1.0	−1.3	−0.9	−1.9	−1.1	−2.7	−1.7

**D) Projected full-year 2021 estimates**								
Projected number of COVID-19 deaths in 2021	456,957		296,341		62,516		75,733	
Estimated 2021 e_x_	76.8	18.4	77.1	18.4	72.6	16.8	78.7	19.5
Reduction from 2018 e_x_ due to COVID-19	−1.9	−1.1	−1.5	−1.0	−2.1	−1.2	−3.1	−1.9

Notes: Apart from life expectancy (e_x_) values from 2018 that are provided by the National Vital Statistics System, all life expectancy estimates are authors’ calculations. Partial-year 2021 estimates and full-year 2020 estimates are based on provisional COVID-19 death counts provided by the National Center for Health Statistics (December 8, 2021 update). Projected full-year 2021 estimates are based on projected COVID-19 death counts through December 31, 2021, provided by the Institute for Health Metrics and Evaluation (November 17, 2021 update).

## Footnotes

1 Because there are reporting delays to NCHS, the number of deaths by December 4, 2021 in the provisional counts is lower than the number of deaths that had occurred by December 4, 2021. Additionally, there may be some misclassification of deaths in 2020 that are attributed to 2021 due to reporting delays. Together, these may lead to an overestimate of the difference in total deaths between the partial-year estimates and full-year projections.

2 At the time of writing, 2018 was the most recent pre-pandemic year for which life tables were available separately by race and ethnicity; we will update these estimates when NVSS releases 2019 life tables. Life tables for other populations, including the Native American and Asian populations, are not published by NVSS at this time.

3 These 2020 estimates differ slightly from our previously published estimates (15), because they include additional deaths that were not yet reported to NCHS at the time of the previous analysis, and they use 2020, as opposed to 2019, mid-year population counts.

4 Not all of these changes should be attributed to the pandemic, however. In particular, although the pandemic likely resulted in much of the upsurge in drug overdose deaths in 2020 and 2021, drug overdose deaths had been increasing during the year prior to the pandemic. Greater availability and use of synthetic opioids in recent years, especially illicitly manufactured fentanyl and fentanyl analogs which are often mixed or used together with other substances and are especially lethal, have led to a greatly enhanced risk of drug overdose (32, 33).
